# Informing the model of care for an academic integrative healthcare centre: a qualitative study exploring healthcare consumer perspectives

**DOI:** 10.1186/s12906-019-2801-4

**Published:** 2020-02-18

**Authors:** Carolyn Ee, Kate Templeman, Suzanne Grant, Nicole Avard, Michael de Manincor, Jennifer Hunter

**Affiliations:** NICM Health Research Institute, Western Sydney University, Penrith, NSW Australia

**Keywords:** Academic health centre, complementary therapies, traditional, complementary and integrative medicine, integrative medicine, integrative healthcare, evidence-based medicine, person-centred care, stakeholder engagement, community-based participatory research

## Abstract

**Background:**

In response to high demand and the growing body of evidence for traditional and complementary therapies, the practice of integrative medicine and integrative healthcare has emerged where these therapies are blended with conventional healthcare. While there are a number of academic integrative healthcare centres worldwide, there are none in Australia. Western Sydney University will soon establish an academic integrative healthcare centre offering evidence-informed traditional and complementary therapies integrated with conventional healthcare in a research-based culture. The aim of this study was to explore healthcare consumers’ views about the perceived need, advantages, and disadvantages of the proposed centre and its relevance to community-defined problems and health and service needs.

**Methods:**

Qualitative methods, informed by community-based participatory research, were used during 2017. Focus groups supplemented with semi-structured interviews were conducted with healthcare consumers. Participants were recruited through paid advertisements on Facebook. Thematic coding, informed by an integrative healthcare continuum, was used to analyse and organise the data. Analysis was augmented with descriptive statistics of participant demographic details.

**Results:**

Three main themes emerged: (i) the integrative approach, (i) person-centred care, and (iii) safety and quality. Participants proposed a coordinated healthcare model, with perspectives falling along a continuum from parallel and consultative to fully integrative models of healthcare. The importance of multidisciplinary collaboration and culturally appropriate, team-based care within a supportive healing environment was emphasised. A priority of providing broad and holistic healthcare that was person centred and treated the whole person was valued. It was proposed that safety and quality standards be met by medical oversight, evidence-informed practice, practitioner competency, and interprofessional communication.

**Conclusions:**

Our findings demonstrate that participants desired greater integration of conventional healthcare with traditional and complementary therapies within a team-based, person-centred environment with assurances of safety and quality. Findings will be used to refine the model of care for an academic integrative healthcare centre in Western Sydney.

## Background

The concurrent use of traditional and complementary medicines and therapies (T&CM) (Table 1) alongside conventional healthcare is common throughout the world. Estimated usage ranges from 9.8 to 76.0% and 1.8 to 48.7% for visits to T&CM practitioners [5]. In Australia, over 70% of the general population have used T&CM, 44.1% have visited one T&CM practitioner [6], and 87% of adults who consult a T&CM practitioner and/or use T&CM will have also consulted a medical practitioner in the previous 12 months [7]. Reasons for T&CM use include promoting health and wellbeing, managing symptoms including side effects from conventional healthcare, and enhancing self-efficacy and agency [5, 6].

**Table 1:** Traditional, complementary and integrative medicine/healthcare

Traditional medicine: is the sum total of the knowledge, skills, and practices based on the theories, beliefs, and experiences indigenous to different cultures, whether explicable or not, used in the maintenance of health as well as in the prevention, diagnosis, improvement or treatment of physical and mental illness [1].	
Complementary medicine: refers to a broad set of healthcare practices that are not part of that country’s own tradition or conventional medicine and are not fully integrated into the dominant healthcare system [1], and may include natural health products (e.g. herbs, vitamins, nutraceuticals), mind-body therapies (e.g. yoga and meditation), and traditional medicine systems (e.g. Ayurvedic, Chinese medicine, and Indigenous healing practices) [2].	
Integrative medicine: refers to the practice of medicine that reaffirms importance of the relationship between practitioner and patient, focuses on the whole person, is informed by evidence, and makes use of all appropriate therapeutic approaches, healthcare professionals, and disciplines to achieve optimal health and healing [3].	
Integrative healthcare: has a broader context than the above, which may be practised by non-medical professionals and is more than preventing and treating disease. Aspects of integrative healthcare include an interdisciplinary, non-hierarchical approach to whole-person care and the promotion of health and wellness [4].	

Despite popular perception that T&CM is natural and therefore safe, its use is not completely without concern with potential direct and indirect risks involved [8–11]. Further, the level of evidence for efficacy and effectiveness of T&CM is mixed. Notwithstanding, some T&CM has amassed moderate or high levels of evidence to support their use [12, 13] and have been incorporated into conventional clinical guidelines globally and in Australia [14, 15]. For example, acupuncture, yoga, and some nutraceuticals are recommended in clinical practice guidelines for pregnancy care, created by the Australian Government Department of Health [16]. Similarly, acupuncture, yoga, relaxation therapy, and hypnotherapy are recommended in clinical practice guidelines developed by Cancer Australia in management of menopausal symptoms for women with a history of breast cancer [17].

In response to high demand and the growing body of evidence for T&CM, the practice of integrative medicine and integrative healthcare (IHC) have emerged where T&CM is integrated with conventional healthcare (Table 1) [18]. Such traditional, complementary and integrative medicine (TCIM) approaches have the potential to enhance patient safety and the appropriate use of T&CM use through mechanisms, such as aiding in the selection of appropriate therapies, minimising the risk of interactions, and ensuring adequate medical oversight [11, 19].

In Australia, TCIM services are largely provided by the private sector in the primary care setting. This includes TCIM services that can be accessed through interdisciplinary clinics [19–21]. There is also growing interest in integrating non-biologically active TCIM services, such as massage/touch therapies, mind-body interventions and acupuncture, into secondary IHC settings [22–24]. However, unlike North America and Europe, there are no university-based academic IHC centres in Australia [25, 26].

In response to this gap, Western Sydney University, Australia plans to establish an academic IHC centre to be housed in the Western Sydney health precinct. The proposed Western Sydney Integrative Health (WSIH) centre aims to be a world-class interdisciplinary academic centre offering evidence-informed TCIM services alongside conventional healthcare for the benefit of the diverse local district. Western Sydney is an area of Sydney that is culturally diverse and contains vast areas of social, financial, and health disadvantage.

With growing attention towards increasing patient and service user engagement [27], we chose community-based participatory research (CBPR) as a democratic method of social inquiry appropriate for implementing improvements to healthcare delivery involving key beneficiaries, such as healthcare consumers and healthcare providers, that would be mutually benefited by the service and inform decision making [28–30]. Rooted in a several-decades long evidence base demonstrating improved health outcomes [31, 32], CBPR has proven successful in engaging socioeconomically and ethnically diverse communities [33, 34]. Considered an important tool in implementation research [29], CBPR may be particularly useful when implementing evidence-based interventions to primary care settings whereby improving access to care and reducing health disparities as well as determining areas of need and establishing priorities for health concerns [35, 36]. At the systems level, CBPR may facilitate sustainable translational research, local health policy, and quality improvement [37, 38]. Indeed, the potential of CBPR to improve intervention effectiveness, encourage equity, and enhance measurement quality [39], including cost-effectiveness [40], are key WSIH priorities.

As part of a CBPR framework, three key stakeholder groups (including healthcare consumers, primary care, and specialist care) were consulted to inform the model of care for our proposed academic IHC centre. This manuscript reports the results of consultation with healthcare consumers in the local community. Responding effectively to patient preferences requires a clear understanding of the way in which patients use and assess clinical services [41]. It is, therefore, necessary to identify patient preferences by including the consumer voice [42]. Little is known about how local healthcare consumers may interact with an academic IHC centre in Western Sydney. The aim of this qualitative study was to explore healthcare consumers’ views about the perceived need, advantages, and disadvantages of the proposed IHC centre and its relevance to community-defined problems and health and service needs.

## Methods

### Design and ethics approval

Qualitative methods, informed by a CBPR framework, were used to collect and analyse data from healthcare consumers. Focus groups [43] were complemented with semi-structured interviews [44] to offer flexibility and gain insights from participants who were unable to attend the focus group sessions. Ethics approval was provided by the Human Research Ethics Committee at Western Sydney University (H12403, 6/10/2017).

### Setting and participants

To increase usefulness and relevance, we sought to recruit a convenience sample of participants in the local district. In alignment with CBPR, this method prioritised the recruitment of healthcare consumers based on ‘local knowledge’ of the community [45] rather than on the need to sample for variation or uniformity [29, 30] so that the synthesis findings get used and have impact.

Participants were recruited through paid advertisements on Facebook (via the Western Sydney University sponsored Facebook page), with targeting of people living in the Greater Western Sydney region aged over 18 years. We endeavoured to recruit participants from a variety of cultural backgrounds; however, due to financial and logistical constraints interpreters were not available. We also attempted to recruit an equal number of participants for each group and ensure uniform representation of gender across groups.

Participants self-selected for the focus groups via the Facebook link, leading to a brief eligibility survey on Qualtrics. Eligible participants were local residents of Western Sydney, able to communicate in conversational English and aged 18 years or over, who had used TCIM services within the preceding 12 months and/or were interested in using TCIM services.

### Data collection

At the time of the focus groups and interviews, participants were provided with a written preamble about the planned IHC service. A brief questionnaire was used to collect anonymous demographic information of participants. Written, informed consent and confidentiality was sought from all participants that included a brief introduction and summary of the aims and rationale of the study.

Experienced researchers (KT, CE) moderated the focus groups and conducted the semi-structured interviews. An 'extended conversation' technique [46] was used with the aim of empowering participants to explore their own context and situation. A semi-structured format (Additional file 1) was used to guide the conversation [44] that aimed to align with what participants perceived as social and health goals. A series of broad open-ended questions across a related range of topics explored: (i) subjective perceptions and preferences regarding TCIM service needs and health-identified concerns, (ii) community-defined gaps and barriers to the provision of TCIM and integration into the proposed IHC service, and (iii) strategic ideas that may be adopted to improve this process (Additional file 1). Data collection continued until data saturation was reached [47] and redundancy in the data was identified [47], whereby the degree to which new data repeated what was expressed in previous data and a full understanding of the participant’s perspective was gleaned. All participants were remunerated with an AUD$25 gift voucher.

### Data analysis

The interviews were audiotaped and transcribed verbatim. KT reread the transcriptions in conjunction with the audio recording to correct any errors. Analysis was based on a hybrid predetermined coding frame and also emerging themes that were first identified by KT using an iterative process of inductive and deductive open coding [48, 49]. This coding process continued until hybrid inductive and a priori thematic saturation was reached in which: 1) there was no emergence of new codes or themes, and 2) identified codes or themes were adequately exemplified in the data, respectively [47]. Combined, this analysis approach served to demonstrate the extent to which the data instantiated previously determined as well as new conceptual categories. We used the continuum proposed by Boon et al. [50] as a thematic tool when analysing data to develop a conceptual framework for describing and comparing different forms of team-oriented IHC practices: parallel, consultative, collaborative, coordinated, multidisciplinary, interdisciplinary, and integrative.

**Fig. 1:**
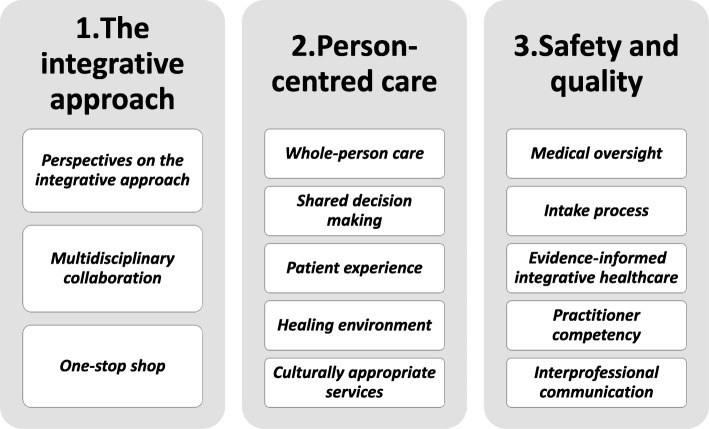
Thematic framework of healthcare consumers’ perspectives on integrative healthcare

Two other team members (CE, SG) then independently coded approximately 20% of the transcripts prior to discussing the thematic results with the whole research team. Coded extracts were then grouped by KT and CE into a thematic map of themes and subthemes and further refined into a number of key themes and subthemes (Fig. 1). Key themes and subthemes were jointly discussed within the research team to increase credibility and trustworthiness of the findings. Themes and subthemes were compared and those that were strongest or common were accepted. The coding framework and narrative summary were then appraised by the whole research team. Consensus decision making was used to resolve any disagreements. As part of providing feedback, participants were involved in cognitive response of the findings.

## Results

**Fig. 2:**
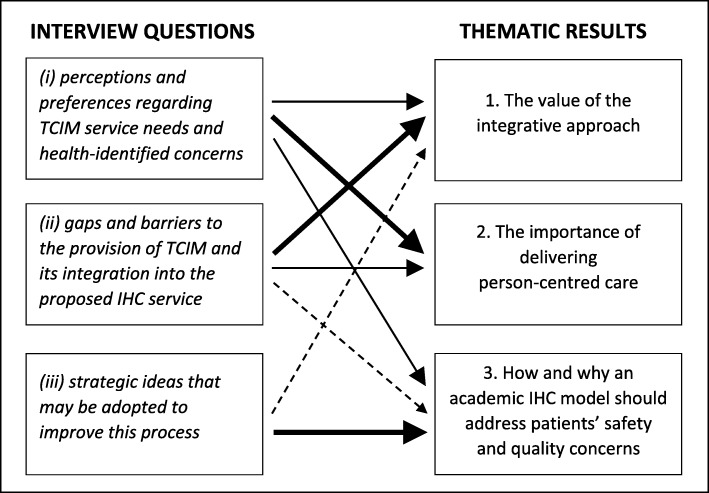
Reconcilement of the semi-structured interview schedule with the thematic results

The results present the findings of two focus groups and two in-depth semi-structured interviews with healthcare consumers during November–December 2017. Three overarching themes were identified: 1) value of the integrative approach for a local clinical service, 2) the importance of delivering person-centred care, and 3) how and why an academic IHC model should address patients’ safety and quality concerns. Three main themes, subthemes, and concepts are discussed within these overarching themes. To illustrate this process, we present a flow diagram (Fig. 2) which articulates what parts of the semi-structured interview schedule (Additional file 1) fed into the thematic analysis. Specifically, the weight and size of the arrow corresponds to how much content from each section of the interview schedule fed into the final theme. Quotations are presented to illustrate participant voices; simple coding has been used to protect anonymity. Additional participant quotes are presented in Table 2.

**Table 2:** Additional illustrative quotes of participants

The integrative approach	
Perspectives on integrative healthcare	
It’s all about taking control of yourself and your needs. I’ve set myself up pretty well and I choose which path to take. That’s how I work now. I am fortunate that I’ve got good practitioners within close range, but I know when I need to look for somebody else or another therapy and I sort of put the feelers out and decide. At the moment I have a great team with me and it all works together (P20).	
One-stop shop	
I don’t have a chronic health condition, but I actually access complementary therapies as part of my health. The people I do use, including my doctor, are very receptive to me doing that. But when I go to my chiropractor, it’s like when are you getting the naturopath here? He’s like I can tell you who to go to, but she’s not there. That’s something that I’ve been looking for more now is that I want to be able to see my naturopath and my chiropractor at the same site (P8).	
I suppose having everything in the one space instead of going to the doctor, this practitioner, that specialist; hydro here, physio there. Having everything that you need in the one space, even stuff you don’t need or you don’t think you need. I might think I don’t need Tai Chi, for example, but really the fact is I probably do to help me with my mental health or whatever or yoga for movement or whatever (P1).	
Person-centred care	
Whole-person care	
I went to my doctor about three weeks ago and he put me on anti-inflammatories, and I’m not supposed to take those because I had a stomach bleed about three years ago, and it’s just not helping, they don’t do anything. I did Pilates and yoga, and I’ve been pain free for the first time in I don’t know how many years with no ill effects (P21).	
I had cancer when I was 27. I’m 52 now. But I just sort of went, no, I’m not going to go the doctor way, I’m just going to do it my own way, because what the doctors gave to me made me really sick. I just had to say well I can’t have that. So I’ve actively done acupuncture for 20 years outside my doctors - for 20-odd years now I’ve had to deal with even my psoriatic arthritis without painkillers, without having treatment and stuff like that (P9).	
Yeah, you go as a rheumatoid arthritis patient, nobody mentioned diet to me. I paid a lot of money for my specialist trying to get good ones, and that was all done by me. Basically GPs only look at you if you’re on medication. There is no one out there that will support my choice to go medication free and work with diet, dah, dah, dah It just becomes stock-standard practice for me … sorry, I don’t have a good relationship with them (P20).	
Shared decision making	
I’ve put a care team together for me. I have rheumatoid arthritis, so I have my care team, which changes. But if I have anybody come in that tells me you cannot cure rheumatoid arthritis, they get the flick before we go any further. I want them to work for me; they support me in what I’m doing. I have a circle that whoever I need to go to at that point in time will give me support. So then that’s what works on a whole like how are they going to work in with my integrative doctor, and work in with me, you know. It’s working with me because I know what my body needs … So it’s all about sharing the decision making and letting me do what I know best about my body (P20).	
Healing environment	
The critical factor in terms of patient care is having that personal relationship; people you trust. If you have that with the clients that come through the door they will come back. They will get more engaged in their treatment. If there's a holistic approach, treating the whole person with the patient at the centre of the care model, people will keep coming back (P7).	
Safety and quality	
Medical oversight	
If the clinician is there overseeing they’re qualifying exactly what you prescribed … We have that assessment done, we actually isolate their condition. We say okay, what are your other needs? What are your limitations as a result of these injuries? How do we get you back moving and engaged? You share and bounce off each other (P2).	
Intake process	
If you have pretty dynamic people on the frontline and they’ve got their list of questions to ask and you can do your ticks and crosses and things when they’re booking. Like the receptionist says can you tell me a bit about the problem? You can work out something there and which way they should go (P21).	
Practitioner competency	
The other issue is practitioners who have been trained in China, so where the expertise in that comes from, so that the patients feel comfort that it was good training, that it was appropriate, that it is as valuable there as what is valued here. They have the same level of expertise. They are now practising here but they’ve gone through all of the hoops (P11).	
I mean I’m really curious about acupuncture, but I think I’d like to know that they’ve had some training first. Like it’s not just some random person sticking needles into me (P18).	

### Participant response and characteristics

Fifty-one healthcare consumers expressed interest in participating and completed the eligibility questionnaire. Participants shared similar characteristics, they were all local, spoke English, and the majority were female and TCIM users. As such, all 51 volunteers were invited to participate in either of the two scheduled focus groups [43]. In November 2017, 19 healthcare consumers participated in one of two focus groups (*n* = 8; *n* = 11, respectively) that lasted approximately 2-h in duration. In December 2017, two participants who initially accepted the invitation to participate in the focus groups (and dropped out on the day) participated in individual telephone interviews that ranged from 50 to 90 min in duration. Table 3 summarises the participants’ characteristics.

**Table 3:** Characteristics of participants

Characteristic	n	%
Age		
18–30	5	26
31–45	7	37
46–60	7	37
Country of birth		
Australia/New Zealand	16	84
India	3	16
Ethnicity		
European/Anglo Saxon/Caucasian	12	63
Asian	5	26
Oceanic first peoples	2	11
Language spoken at home		
English	18	95
Other	1	5
Gender		
Female	16	84
Male	2	11
Other (“born female but always wanted to be male”)	1	5
Education		
High school - Year 12	1	5
TAFE or vocational college	3	16
Bachelor degree or higher	13	68
Missing	2	11
Employment status		
Employee	7	37
Self-employed	4	21
Home duties/caring for children or family	1	5
In education (e.g. going to school, university)	3	16
Doing voluntary work	1	5
Unable to work because of illness	1	5
Missing	2	11
T&CM use (*n* = 16)		
Vitamin and mineral supplements	12	75
Nutritional supplements (e.g. fish oil, antioxidants)	11	69
Massage	11	69
Acupuncture/acupressure	9	56
Yoga	8	50
Meditation	7	44
Relaxation techniques	7	44
Chiropractic	5	31
Chinese herbal medicine	4	25
Prayer	4	25
Tai chi	3	19
Naturopathy	3	19
Spiritual healing	3	19
Osteopathy	2	13
Homoeopathy	2	13
Reflexology	2	13
Qigong	1	6
Bowen therapy	1	6
Other	1	6

### Theme 1: the integrative approach

Study participants spoke of the perceived benefits of an integrative approach to healthcare. Perspectives on TCIM and subsequent IHC and its delivery mostly appeared to align with a coordinated team-based healthcare model, with perspectives falling along Boon’s continuum from parallel and consultative to fully integrative, non-hierarchical models of healthcare [50]. Such broad collaboration would engage patients as partners in addressing the different reference points that shape patients’ concept of health to create a healing environment that was less medical.

#### Perspectives on the integrative approach

Participants commented that *“nothing works in silos”* (P11), and the combined approach of TCIM and conventional healthcare promotes *“preventative medicine”* (P1) and provides better care than either approach alone as *“they’re working together rather than against each other”* (P13). Further, it was suggested that an integrative approach to healthcare promotes safer care as *“everyone* [is] *working together safely in the system”* (P14).

By and large, participants viewed TCIM as a wide range of health interventions ranging from *“prevention to treatment”* (P8) to “*rehabilitation and recovery”* (P1). Participants conceptualised TCIM as *“multiple modalities of care*”, not just usual care, but also “*unconventional care”* (P3) that helps patients manage, maintain, and *“restore health”* (P10). The intensity of care, as well as the therapies/support mobilised, was tailored to the participant’s need and risk and moderated by personal preferences.

*The main thing here is we’re choosing to actually help ourselves. I have a GP* [general practitioner] *and a specialist, but I actually then attach myself to other things as well like complementary therapies which make me feel better. That is part of the whole thing. We need to feel better about our bodies and where we’re moving through* (P9).

Participants noted that TCIM provided *“heaps more choice than conventional stuff”* (P21). Rather than viewing TCIM as an ‘alternative’ to conventional healthcare, it was thought that an IHC centre would give patients more informed management options.

*Whenever I do go to the doctor it’s an opportunity often missed because there isn’t that other side of things that are being offered. So for me, opportunity often lost, for which this model would certainly be more useful* (P15).

Since you *“can’t put people in cotton wool”* (P8), an emphasis on prevention-oriented care was frequently discussed along with practitioners being proactive in their holistic approach to optimise health.

*It would be great* [holistic care] *because that would stop my 25 year journey. I almost felt like I was doing my own preventative medicine. I wasn’t supported in my preventative medicine because actually doing things like acupuncture, Chinese herbs, yoga, mindfulness, meditation is totally separate definitely to GP stuff … They* [GPs] *need to know they’re going to give us care and have an understanding about our needs … We want to last a few more years rather than die quick* (P9).

#### Multidisciplinary collaboration

Participants felt that this broad, holistic view of health and wellbeing is not well acknowledged by conventional healthcare, thus articulating an integrative approach that encourages care coordination, team-based care, and patient engagement.

*Often people with a chronic condition have multiple chronic ones … So, the fact that there is a team who understands all those conditions and then the complementary medicines that can also assist you treating the whole body* (P15).

*You know the whole system approach is a much better approach than we all just get sick and get shoved in a hospital and then get shoved out and forgotten about and die very young. It’s a sausage factory now. That’s got to stop. This is one method of looking at it differently and saying well let’s not just move people through a conveyer belt* (P11).

Further, participants articulated the concept of an integrative approach bringing the two sides of healthcare (namely TCIM and conventional healthcare) closer together and working as a team instead of in parallel. This was thought to differ from prevailing patterns of conventional healthcare that are often compartmentalised, fragmented, and delayed. Many participants perceived that *“quite an impressive reputation could be developed if there’s multiple respect amongst the disciplines for one another”* (P11). It was agreed that this teamwork needed to happen in both directions, with the application of the best of conventional healthcare and TCIM. For some, a parallel model where practitioners practise under the one roof would miss the mark.

*You need to offer me doctors and other people that have an interest and knowledge of these alternatives and can work together, including specialists who acknowledge these alternatives. Otherwise if you have a stock-standard GP you’re just another doctor’s clinic. I wouldn’t come down. I’d go, yeah, that’s a token effort, good on you, but not for me* (P20).

#### One-stop shop

As part of true integration, participants spoke of the convenience and benefits of a *“one-stop shop”* and being able to deal with health issues *“holistically in one space”* (P13). Participants thought that this concept would ensure the full spectrum of IHC and prevention opportunities are included in the care delivery process. In particular, participants thought that *“keeping everything cohesive”* (P9) represented *“good continuity of care”* (P21) rather than making *“5000 trips all over Sydney to see people”* (P13). Participants favoured the idea of an *“allied network”* (P2) and opportunity of seeing everyone within the *“holistic clinic model”* (P2).

*Having that one-stop shop and continuing care where everything is all there available for you so you don’t need to take extra time off work or whatever, especially if you’re available after hours. That’s a big plus* (P1).

### Theme 2: person-centred care

For participants, the ideal orientation of healthcare was person centred. From the perspectives of our participants, person-centred care took into account differences in individual conditions, needs and circumstances, patient preferences and values, and shared decision making, as well as providing a holistic, whole-person approach to health and healthcare.

#### Whole-person care

Participants described a care process in which patients and caregivers work together to foster seamless engagement of multiple health domains (e.g. physical, psychological, cognitive, and social) and aims (e.g. therapeutic, preventive, health optimisation). This involved matching a variety of interventions, both conventional and TCIM, with the unique problems and preferences of patients that may include issues not well understood by conventional healthcare.

*Traditionally GPs will look for a medical, physical solution, particularly with issues around mental health. It’s about looking at that more holistic view if there’s a physical ailment but where it might be coming from - it’s here in my arm but actually it’s a referred pain or it’s to do with my gut* (P13).

*The average Joe may just want massage. They may just want acupuncture or yoga. But there may be other presenting issues that you can tap into. There might be underlying depression or anxiety or there could be a pain issue or a chronic back condition. That way you tap into that and you provide the holistic service. That’s what wellness is about (P8).*

Participants also expressed the desire for healthcare that “*emphasises caring for you as a whole person*” (P12). Likewise, participants expect their therapists to approach them with a holistic approach that integrates *“the whole aspect*” (P7). It was thought that this process would ensure optimal health and wellbeing, and therefore, build a successful service.

*It looks after the mental wellbeing, the physical wellbeing, the emotional wellbeing, and also the social wellbeing … If there’s a holistic approach, treating the whole person with the patient at the centre of the care model, people will keep coming back* (P7).

#### Shared decision making

Shared decision making (SDM) was considered an important aspect of an integrative, collaborative approach. Participants sought to be explorers of their own health, making decisions in partnership with various providers. Participants also described a desire to discuss TCIM use openly with providers without being dismissed or not taken seriously. They described being rushed, not being listened to, and “*worrying about the eye-rolling and being judged as silly*” (P14).

In this context, teamwork reflected the desire for a care process where the patient is actively contributing as a team member and their decisions are respected. As one participant noted: *“I want a team who supports my healthcare choices”* (P20). Better access to IHC, and a willingness to pragmatically seek out shared opportunities, were also considered important concepts.

*I decide what the best therapies are for me, but I usually run it by my GP first and we decide together what would work. Even though I exercise choice, my integrative GP has a say and I listen to her. But she lets me decide too, and that’s how I prefer to do things. I wouldn’t have anything if I thought it would interfere with what I’m having or already doing with her* (P21).

#### Patient experience

Central to person-centred care was the patient experience. Participants emphasised that *“finding the right people to become part of the team”* (P20) was important and this “*starts at the front desk”* (P1). Within this concept, it was thought that practitioners must not only be competent, they must have the right mindset that aligns with the core values of IHC. Further, since *“these things cost a lot of money”* (P20) patient experience also appeared to influence demand for IHC services.

*It starts with the receptionist. It’s like if you don’t get a smile and a hello and it’s just give me your Medicare card, it’s like see you later. It’s a team environment, everyone is entitled to that. People will benefit from that and be willing to come back and pay for it. If you can get a person on a path to wellness they’re going to be more able to engage in meaningful work and meet all of their health domains* (P7).

#### Healing environment

Participants anticipated that in contrast to conventional healthcare services, the environment of an IHC centre would be pleasant and supportive so as to facilitate healing. Important to the centre’s environment were key concepts of the provision of *“comfort”, “nice smell”, “nice surroundings”* (P20), and *“you walk in and it’s not clinical”* (P20).

*You go and sit in these clinical rooms with black lounges and they don’t give you the right information, there’s no support, it’s an awful environment. It’s no more comforting than flying to the moon* (P20).

Within the practitioner-patient dynamic, a commonly identified concept expressed by participants was a caring and passionate practitioner-patient alliance at care entry points to improve patient engagement and support. In their interactions, participants reiterated that practitioners must be able to develop therapeutic relationships with the people they care for, gain the trust and *“mutual respect”* (P18) of patients by communicating empathically and professionally, and be able to *“work directly with the patient”* (P2) to offer *“something extra”* (P8).

*As soon as you walk into that place and they engage you it’s either a yay or a nay as to how you feel about the place. For it to be effective everyone has to buy in; there has to be complete buy in. You really have to have sympathetic and passionate staff who know what they’re doing for it to work* (P8).

Participant perspectives also described dissatisfaction with their experiences within conventional healthcare.

*Most people will exhaust the general system looking for someone to listen to them and treat them like a normal human being* (P10).

#### Culturally appropriate services

Within the core values of an IHC service, participants discussed the concept of non-judgemental, inclusive attitudes towards TCIM and cultural competency. For some participants, the importance of TCIM was perceived as part of embracing patients’ cultural differences and beliefs and the need to provide culturally sensitive care, which was considered particularly important in the diverse cultural community of Western Sydney. Notably, however, providing TCIM services that matched a patient’s cultural background was not a prerequisite for providing appropriate services.

*I know Aboriginal people from the Northern Territory who go to acupuncturists because they trust them, there’s an understanding of culture and a sharing of knowledge* (P9).

### Theme 3: safety and quality

In keeping with the integrative approach and person-centred care, participants highlighted that safety and quality are key considerations for IHC services at every level: regulatory bodies, service providers, health professionals, and lay-people. There was the expectation that an academic IHC centre would have high standards and sound clinical governance. Further, it was anticipated that the integration of conventional healthcare and the Western scientific approach with T&CM would minimise potential risks by ensuring there were necessary checks and balances in all aspects of service delivery and communication within and outside the centre. This, in turn, was considered important in supporting patients to make informed decisions. Whilst there was support for medical oversight from a GP, participants held different views about whether processes, such as intake, should be GP-led or collaborative.

#### Medical oversight

Within the theme of 'quality and safety', participants’ understanding of the concept of 'medical oversight' primarily involved the need for a *“level of clinical governance”* (P11) and structure *“to make sure that as a team you’re not missing anything” … “it’s a bit like a safety net”* (P15). Importantly, participants wanted to feel *"safe"* (P11), with a *“level of expertise”* (P16) to oversee such things as credentialing practitioners and therapeutic decisions that would ensure safe clinical care and “*reassurance about minimising risk”* (P15). Identification of safety issues and *“red flags”* as well as perspectives of appropriate guidance of care, for example, *“tier one, two, three and four”* (P2) was also highlighted. Further, it was thought that medical oversight would ensure that *“if it needs to get referred up the chain you’ve got that traditional model to fall back on”* (P2).

*I can see absolutely where that’s a risk and you need a gatekeeper like a GP … the very fact that these practitioners all co-exist with a GP and it is operated by a university clinic actually gives people some of that reassurance* (P13).

#### Intake process

It was perceived that, as part of a coordinated whole-of-centre approach, a thorough central intake process was necessary. Participants were asked about a GP-led intake and triage process. Some thought that *“GPs could play a really significant role with regard to safety”* (P13). For the initial assessment (particularly if they were not already under the care of a GP), participants thought that patients would probably need to see a team GP for the main purposes of *“those important check offs, preventative measures, and checks and balances”* (P2), and informing patients about appropriate therapies that they may otherwise not consider using.

*If you’ve got someone coming in with specific problems they can be assessed by a GP so that you don’t have someone do a yoga class that’s got issues they haven’t really discussed, the right questions haven’t been asked, and they do more damage or something like that* (P11).

*The doctor can assess and see what’s happening. I might come in saying I want acupuncture and if I was assessed they might go you know what, acupuncture is good but you might find this works even better for your specific problem. We can be guided in the right direction by someone that knows the services you’re offering* (P13).

Conversely, within some participant perspectives, whilst checks and balances were important, a GP as the gatekeeper was considered unnecessary and potentially contradicted the integrative approach. Rather, such participants emphasised a coordinated, whole-person approach whereby *“the process is more about the whole practice coming together to develop an intake process”* (P14) so that *“everyone gets an understanding of that triage role”* (P11). As one participant pointed out, *“that’s a more collaborative way of identifying the right practitioner”* (P14). For this reason, it was considered that within the boundary of safety “*different pathways”* (P18) and *“multiple entry points”* (P14) would be useful.

*So someone comes in and says, I’ve got a headache, well you need to talk to so and so. As things get talked on do they then get referred to Chinese massage for instance or this or that as opposed to you as the patient going oh well normal medicine hasn’t worked - now I’ll try this thing or do I do that thing. So you need those different pathways within the practice* (P18).

Participants perceived that, “*as part of a holistic patient care system”* (P7), a thorough central intake process was necessary to “*really capture what’s happening”* (P15). That way, *“if it raises any flags they can say, okay, that needs to be checked out”* (P14) and *“flicked to the next port of call”* (P2).

*If you have pretty dynamic people on the frontline and they’ve got their list of questions to ask and you can do your ticks and crosses and things when they’re booking* (P21).

#### Evidence-informed integrative healthcare

Participants highlighted the importance of incorporating scientific evidence about safety and efficacy to inform decisions about healthcare.

*I’m all for evidence based, you don’t want the total opposite at the other end of the spectrum. You want to know that what’s assisting you has some solid science behind it* (P11).

Participants noted that *“there’s a pre-conceived idea that complementary medicine is a quack* [and] *a lot of it is de-valued and pooh-poohed by the medical profession”* (P1). While participants valued the therapeutic benefits and philosophies of TCIM, for many, it was considered important *“to prove that the centre has experience and that the whole premise is based on evidence”* (P11) as this would increase perceived legitimacy and reassure patients that they are receiving appropriate healthcare.

*To have a place of authority that has looked at the evidence behind practices in a holistic manner is really beneficial, because it means that people can recognise that that’s got some credibility* (P7).

#### Practitioner competency

Within the context of safety, the concept of practitioner competency was a recurring theme. Participants emphasised the importance of high and uniform standards of clinical practice and assurances that practitioners *“had some training first”* (P13), were “*certified in their own field”* (P18), and stayed within their scope of practice in order to minimise safety concerns.

*He’s fully trained within that scope so straight away he knows what he’s doing. Every training he’s had since has been what is the most accredited or appropriate, so there really is that legitimacy* (P13).

When choosing TCIM practitioners for the centre, participants emphasised the importance of engaging credible practitioners that comply with professional, ethical and practice standards, act as responsible agents for patients, and have a sufficient level of clinical competence and accountability.

*You need quality assurance, like the umbrella of clinicians all representative of the yoga community that you hold up as a peer. You’ve got to have qualified people* (P2).

Within this concept, choices regarding TCIM services were, in part, based on regulatory status of practitioners and associated level of expertise so that “*it’s not just some random person sticking needles into me”* (P18), “*tailoring the practitioners from particular associations that are relevant* [so patients] *get recommended to the appropriate person”* (P13), and *“so patients feel comfort that it was good training, that it was appropriate*” (P11).

Several participants favoured IHC services with practitioners who had “*cross-training”* (P12), such as TCIM practitioners with experience working in conventional healthcare environments and dually-trained medical or allied health practitioners. At the very least, participants perceived that medical practitioners should *“be more engaged and informative about these things* [TCIM]*”* (P20) and have *“an interest in the other side”* (P16).

#### Interprofessional communication

As part of routine checks and balances, as well as delegation of care, communication and reporting systems were considered a key feature of integrative care. Specifically, participants wanted to keep their regular GPs and other healthcare providers up-to-date about TCIM management so that the patient focus is supported.

*… a written assessment at the beginning of what you think you can do to help … Then at the end of the six weeks probably how things are going and options of ongoing treatment. Maybe from that every few months just something saying how the ongoing things are going or a letter saying everything’s gone really well* (P21).

Along with the concept of strong teamwork among practitioners in an IHC centre, participants also discussed the necessity of *“having a good referral network outside”* (P6) of the WSIH centre, and that integration and effective teamwork with the wider healthcare environment would promote safer healthcare. Within this concept, participants highlighted coordinated care, which aligns with the integrative approach. Herein, participants recognised the integral role of GPs in the Australian healthcare setting, and the need for medical practitioners outside of WSIH to have access to “*a centre with many practitioners that offer alternatives to refer people to”* (P20).

*The GP will sort out what they can sort out first. I’m really happy to go to a GP that’s happy to say well you know what, I’ve gone about as far as I can go, I think you should see this* [TCIM] *person or that.* (P21).

In this vein, participants discussed a bi-directional referral process from their regular GP so that additional care options across the relevant life dimensions could be sought yet *“continuity of care*” (P21) maintained. *“That way, it’s not just about GPs learning about alternate therapies, it’s about alternate therapies developing another respect for GPs back again”* (P14). The perceived goal among participants was for GPs external to the centre to work collaboratively with a team of TCIM experts to promote interprofessional collaboration.

## Discussion

This qualitative study provided a unique opportunity to engage local healthcare consumers in order to understand their perspectives to better inform the model of care for WSIH and IHC best practice. Healthcare consumers in this study saw value in offering a diverse range of therapies through a centre, such as WSIH, due to the perceived benefits of accessing high-quality TCIM services in a single location. By integrating conventional healthcare with T&CM, rather than using them as standalone therapies, strengths and weakness of the different therapeutic approaches would be complementary and benefit patients who sought to access a range of modalities to address their holistic healthcare needs. Such findings are not uncommon, as healthcare consumers often perceive that the combination of conventional healthcare and T&CM is better [51–53], safer, and more effective [54] than either approach alone.

The way in which integration of T&CM and conventional healthcare should be delivered is being explored both theoretically and in many emergent practices [50, 55–57]. Consistent throughout these definitions and perceptions, however, is a desire from healthcare consumers to experience the best of both worlds, as expressed by our study participants who held various IHC concepts that spanned a continuum of intensity. This continuum reflected the conceptual framework originally conceived by Boon et al. [50]. In this framework, as the healthcare consumer moves from left to right on a continuum, there is a wider range of disciplines with increased collaboration between practitioners and healthcare providers, less hierarchy, and greater patient involvement in decision making. In our study, participants expressed a range of ideals that spanned along the continuum, but all went beyond simply being provided with parallel or consultative practice. In general, participants conceptualised collaborative care as a minimum requirement of the integrative approach. Teamwork and collaboration was emphasised, not just within the centre, but more broadly within the healthcare landscape that included the patient’s usual GP and medical specialist(s) as part of person-centred care where the patient was an active participant in the IHC team.

A key desire for integrating T&CM and conventional healthcare focused on assurances of safety, which encompassed practitioners within an IHC centre providing appropriate guidance and information about TCIM and presenting health conditions. A recent review exploring Australian consumer needs and preferences for healthcare safety and quality (including TCIM) found that patients like to be informed about the potential benefits and harms of different options available to assist with decision making regarding care and treatment pathways [58]. Consumers needed information on a range of topics spanning the patient journey, including information about specific medical conditions and their management, treatment and prognosis, and information regarding health professionals and their various roles [58].

The paradigm of person-centred care fits well with IHC as it emphasises SDM and preference-based medicine, fosters partnerships with patients, and integrates T&CM with conventional healthcare thereby increasing therapeutic options [59]. The evidence for SDM suggests its benefit as an enabler for effective provider-patient communication that, in turn, may support IHC delivery [60, 61]. Studies have shown that communication, the patient-provider relationship, and preference-based care are important dimensions of person-centred care and SDM [62], and that strategies designed to increase SDM may have a positive impact on patient satisfaction [63] and health outcomes [60, 61]. However, other dimensions of person-centred care, such as comprehensiveness of services, cultural sensitivity, and whole-person care, are not well researched [64]. Findings from our study suggest that, along with fostering SDM, IHC services may further support person-centred care by offering a diverse range of TCIM therapies that are culturally appropriate and holistic. Our participants also clearly valued the role of scientific evidence within the process of SDM.

Further, while participants appeared to embrace the benefits of conventional healthcare, there was a strong sense that health was more than just the absence of disease, and a holistic approach that takes into account emotional, social, and physical wellbeing, was important to them. More specifically, participants wanted a person-centred approach (including SDM) that met their individual needs, articulating frustrations with current models of care from previous healthcare experiences. The principles underpinning person-centred care reflect autonomy and self-determination [65]. In this, the patient brings experience, circumstances, and values to the consultation, and the health professional brings knowledge, diagnostic, treatment, and outcome possibilities [66]. While person-centred and whole-person care are not unique to IHC, our study participants described experiences in conventional healthcare that undermine the principles of person-centred care, such as being judged negatively and having nobody (within conventional healthcare) who was willing to support their choices to use TCIM approaches*.* Therefore, attending to the principles of person-centred care may not necessarily reflect anything unique about IHC, but remains a vital component in order to provide the model of care that consumers desire. Person-centred care is not simply a matter of patient preference and experience. Systematic reviews show that person-centred care results in increased adherence to management protocols, reduced morbidity, and improved quality of life for patients [67]. Therefore, improving health outcomes may need to attend to principles of person-centred care regardless of the level of integration with TCIM.

An important strength of conventional healthcare, however, was that it brought greater assurances of safety and quality. Participants in our study generally valued medical oversight and many wanted formal communication and referral pathways between their regular GP and the centre. However, this did not always equate to wanting a GP-led intake process at the centre. Like other research, our findings reflected the heterogeneity in patient preferences. For instance, a recent Australian survey of healthcare consumers identified that the preference was often for triage by a non-medical staff member [53]. Other studies have found that patients also wished to be explorers of their own health, capable of deciding for themselves among various TCIM and conventional healthcare services [68]. In an exploration of IHC centres in the US, whilst triage was often undertaken by the medical practitioner, a nurse or patient navigator was also well received by patients [69]. As a result, it may be that the triage process is best guided by patient preference, level of integration required, and complexity of the presenting health condition.

The importance of medical oversight and safety concerns was interrelated with the competency and credentialing of practitioners. Participants highlighted the importance of minimum standards for expertise and experience amongst practitioners. Indeed, other local research with cancer survivors affirms that a perceived advantage of IHC is the extra reassurance it offers regarding the quality and credentialing of TCIM practitioners [70]. Such concerns are not unwarranted as minimum training requirements, qualifications, professional standards, and the regulation of TCIM practitioners vary significantly across the various professions in Australia and throughout the world [71].

There are inherent tensions between TCIM and conventional health care that are likely to arise when attempting to integrate different professional cultures and paradigms [21, 72–74]. Along with various reasons given for and against a GP-led model verses a more collaborative model, participants anticipated that a university-based clinic would vet the scientific evidence of the TCIM therapies provided. This was not only important for the safety of patients but was needed to help enhance the legitimacy of TCIM with the dominant medical profession. It is common for patients in Australia who use TCIM to express feeling stuck in the middle between TCIM and conventional healthcare practitioners and left having to justify their choice to use TCIM [70, 75]. The extent to which IHC models, such as the one proposed by WSIH, might advocate on behalf of their patients was not fully explored during the interviews, nor were potential professional conflicts discussed. Managing medical dominance, however, will be important for WSIH and affirms the broader CBPR engagement strategy that includes a wide range of TCIM and conventional healthcare practitioners.

This study has several strengths and weaknesses. Strengths are that our study was informed by CBPR, an appropriate research orientation for implementing improvements to healthcare delivery involving beneficiaries across select stages of the research process [28–30] that will be mutually benefited by the intervention. The findings from this study, along with the findings from qualitative inquiry from two other major stakeholder groups (namely primary care practitioners and medical specialists), will be used to inform the development of WSIH and its model of care. Recognising the community as a unit of identity, and building on the strengths and resources of the local community, participants became key informants who contributed important knowledge to the course of this research that informed decision making [30]. Indeed, such an approach fits well with IHC and its emphasis on consumer engagement, person-centred care, and SDM [59]. To our knowledge, this is the first qualitative study in Australia to explore local needs for an academic IHC centre. The final model of care will be discussed in a subsequent manuscript.

However, although we aimed to recruit participants who were non-TCIM users as well as TCIM-users, the vast majority of our sample had used CM within the last 12 months. Therefore, we have not been able to capture the attitudes and experiences of consumers who had less exposure to TCIM. Our sample was also largely female, well-educated, and Caucasian bar a small number of participants who were born in India. Whilst the demographic sampled reflects TCIM users in Australia [6, 76], it was not representative of the local demographics that show well above the national averages for many CALD groups with Chinese, Indian, Lebanese, Korean, and Vietnamese being the most common [77]. Other research in the local area has explored the views, experiences, and IHC preferences of non-English speaking cancer survivors from Chinese, Vietnamese, and Arabic backgrounds [70]. The findings were complementary, and affirm the need for more IHC services in the region that respect traditional healing practices and are culturally appropriate, accessible, and affordable. Given that a key aim of WSIH is to serve its diverse community, ongoing community engagement with a greater focus on different local CALD groups, including Aboriginal and Torres Strait Islander communities, is important.

## Conclusions

In our qualitative study, we engaged local healthcare consumers to inform the model of care for an academic IHC centre in Western Sydney, Australia. Participants called for greater integration of TCIM with conventional healthcare, ranging from a collaborative model to fully integrative according to the individual patient’s needs and preferences. They identified priorities of providing safe, high-quality IHC that treated each patient as a whole person. These findings will be incorporated into the design, implementation, and evaluation of Australia’s first academic university-based IHC centre in Western Sydney.

## Supplementary information

**Additional file 1.** Interview schedule

## Data Availability

The datasets generated and analysed during the current study are not publicly available due to ethics approval for use of the data in this study only.

## Abbreviations

CBPR: community-based participatory research
GP: general practitioner
IHC: integrative healthcare
SDM: shared decision making
T&CM: traditional and complementary medicines and therapies
TCIM: traditional, complementary and integrative medicine
WSIH: Western Sydney Integrative Health
