# Supplementary material for: Informing the model of care for an academic integrative healthcare centre: a qualitative study exploring healthcare consumer perspectives
Source: BMC Complement Med Ther. 2020 Feb 18;20:58. doi: 10.1186/s12906-019-2801-4 (PMC7076816; doi:10.1186/s12906-019-2801-4)
Supplement: Supplementary file 1 — Additional file 1. Interview schedule [file 12906_2019_2801_MOESM1_ESM.docx]

**Appendix 1:** Interview schedule

# **Focus group and interview schedule: The local community**

Brief introduction, rationale of the project, and overview of the prospective Western Sydney Integrative Health (WSIH) centre and services to be provided

Explain to participants that they had been invited as representatives of their community, and emphasise the importance of their “voice” in participation in addressing the issues outlined in the discussion

The aim of this project is to examine the subjective experience and meaning from the community perspective, and perceptions about need for traditional, complementary, and integrative medicine (TCIM) therapies and integrative healthcare (IHC) service delivery in Western Sydney, with the purpose of informing future service delivery and resource development for the prospective WSIH centre.

**This research focuses on *integrative healthcare* (i.e. blending of evidence-informed TCIM therapies with conventional healthcare)**

A $25 e-Gift Card will be emailed to your nominate email address

**Qualitative schedule**

1. In general, what do terms such as ‘traditional, complementary, integrative, alternative, IHC’ mean to you?

- What term(s) do you specifically associate with TCIM services?

***Prompt terms:*** ‘integrative healthcare, wellness, wellbeing, traditional, holistic, complementary, natural, restorative, supportive care’

- What is your expectation of wellness, what it means, conceptual understanding
- What terminology do you feel is most appropriate to the centre? Why?

1. Non-TCIM users:

- Have you ever been interested in or wanted to use these types of services?
- If yes, what prevented you from using them? If no, why not?

***Prompts:*** unsure if it would help, unsure about who to consult, cost, unable to find someone nearby, doctor/friend/family advised against it.

1. What TCIM services are you aware of in the local area?

- How did you hear about them?
- How adequate are these current services in meeting the needs of the community?
- Do you currently face any barriers to accessing TCIM services?
- What would you like to see more of in Western Sydney in terms of TCIM therapies? WHY?
- Have you been to any centres that combine TCIM and conventional healthcare? What did you think of the service?

1. What do you think the advantages and disadvantages are of combining TCIM and conventional healthcare?

- How do you think we can work together to help you achieve better health?

1. If there was a local IHC centre, how could we safely provide a service that you are satisfied with?

- What are your expectations for local TCIM services
- What would give you confidence in using our service?
- How would you decide if the practitioner is credible and experienced?
- What sort of services/therapies would meet your healthcare needs?
- Where or how would you like to access these services?
- Would you like your treating doctors to know about our service?

***Prompts:*** linking patients to appropriate services, access to TCIM, seamless integrative care, credentialing, clinical training, safety protocols, GP awareness/education.

1. What sort of health problems would you consult our centre for?

***Prompts:*** general wellbeing, women's health, preventive health, pain management, mental health, cancer supportive therapies, survivorship, unexplained symptoms (e.g. fatigue/chronic fatigue syndrome), chronic disease, neurological problems

1. How would you like to see this service operating?

- Do you think that patients should be referred to the centre by their GP or another non-TCIM health professional? Or should patients self-refer?
- Do you think that patients should have an initial consultation with a non-TCIM healthcare professional like a GP/other medical doctor or nurse?
- What would you be willing to pay/patients in the region be willing to pay for: integrative GP consultation (short consultation 15 minutes/long consultation 45 minutes); acupuncture treatment; yoga therapy; yoga/tai chi classes; oncology massage
- How should we communicate with your usual treating doctor(s), and how often?
- Do you think medical doctors should be onsite at the centre? If so, what type of medical doctor, what role should they play, how many doctors?

***Prompts:*** referral, do they want self referral, do they want written communication?

1. Do you think our centre should offer comprehensive primary care as well as TCIM (i.e. when needed, we can provide all the services that a GP provides such as screening, preventive activities (e.g. Pap smears, immunisation), and acute care?

- What other services do you feel we should provide?
- Should we also have specialists in the centre?
- Should we have allied health practitioners at the centre (e.g. dietitian)

1. How should we advertise our services?
2. What might be the barriers and facilitators of successful implementation of this service?

- Would there be any problems with establishing an IHC centre (e.g. would we face resistance?)
- How can disadvantages be addressed to suit your healthcare needs?

1. Is there anything else you feel is important to add or highlight?
2. Would you like to receive a copy of the results?

- If yes, please provide email address details

1. Would you be interested in continued design of the model of care for our centre? This may include volunteers to be involved in a steering committee or participation in research activities?

- Please provide email address details
